# Exploring Automated Classification Approaches to Advance the Assessment of Collaborative Problem Solving Skills

**DOI:** 10.3390/jintelligence10030039

**Published:** 2022-07-04

**Authors:** Jessica Andrews-Todd, Jonathan Steinberg, Michael Flor, Carolyn M. Forsyth

**Affiliations:** 1 Learning and Assessment Foundations and Innovation Center, Educational Testing Service, Princeton, NJ 08541, USA; mflor@ets.org (M.F.); cforsyth@ets.org (C.M.F.); 2 Foundational Psychometric and Statistical Research Center, Educational Testing Service, Princeton, NJ 08541, USA; jsteinberg@ets.org

**Keywords:** collaborative problem solving, collaboration, assessment, automated annotation, machine learning, cluster analysis, skill profiles

## Abstract

Competency in skills associated with collaborative problem solving (CPS) is critical for many contexts, including school, the workplace, and the military. Innovative approaches for assessing individuals’ CPS competency are necessary, as traditional assessment types such as multiple -choice items are not well suited for such a process-oriented competency. In a move to computer-based environments to support CPS assessment, innovative computational approaches are also needed to understand individuals’ CPS behaviors. In the current study, we describe the use of a simulation-based task on electronics concepts as an environment for higher education students to display evidence of their CPS competency. We further describe computational linguistic methods for automatically characterizing students’ display of various CPS skills in the task. Comparisons between such an automated approach and an approach based on human annotation to characterize student CPS behaviors revealed above average agreement. These results give credence to the potential for automated approaches to help advance the assessment of CPS and to circumvent the time-intensive human annotation approaches that are typically used in these contexts.

## 1. Introduction

Many of the activities that we engage in during our everyday experiences involve interacting or working with other people, such as working with classmates to complete a project, working with engineering colleagues to design a product, meeting with business partners to resolve an issue for a client, or family members coming together to determine the optimal location for a family vacation. Recent technological, health, economic, and social changes and challenges (e.g., the COVID-19 pandemic, climate change, rising income inequality, ethics for artificial intelligence) have only exacerbated changes in the way we live, work, and learn, creating additional demand for individuals to develop capabilities associated with collaborative problem solving (CPS). Specifically, many of the challenges we face today require teams of individuals to come together to work on finding solutions. Indeed, many employers, organizations, and government agencies have deemed CPS and related constructs as critical for career and workplace success in the 21st century (Fiore et al. 2017; McGunagle and Zizka 2020; OECD 2013b; Partnership of 21st Century Learning 2016; Whorton et al. 2017). This has garnered increased interest in multiple contexts (e.g., K-12 education, higher education, workplace contexts) in the assessment and development of CPS skills. One important consideration for assessing and developing CPS skills is understanding and optimizing approaches for identifying and reporting what individuals know and can do with respect to CPS capabilities. In the current study, we describe computational linguistic methods for automatically characterizing students’ display of CPS skills and compare these methods to often-used human-driven approaches. Such an examination can help advance CPS assessment work to circumvent the time-intensive nature of traditional human annotation methods.

### 1.1. Current Approaches for CPS Assessment

Collaborative problem solving involves individuals working together by sharing information and pooling their knowledge and effort to reach a solution to a problem (OECD 2013b). The behaviors that individuals engage in during collaborative problem solving involves a social dimension associated with collaboration and teamwork types of behaviors and a cognitive dimension associated with problem solving and taskwork kinds of behaviors (Andrews-Todd and Forsyth 2020; Hesse et al. 2015; OECD 2013b). As such, CPS involves a complex set of skills across multiple disciplines (e.g., linguistics, computer-supported collaborative problem solving, individual problem solving) (Care et al. 2016), adding to the complexity of measuring skills associated with CPS. The complexity is magnified by humans collaborating in natural language conversations with one another creating an exponential number of discourse moves to capture numerous aspects of the dimensions of CPS.

The complexity of CPS and its process-oriented nature make the use of traditional kinds of assessment (e.g., multiple choice questions) not well-suited for capturing evidence of individuals’ CPS skills (Davey et al. 2015). This has led to the development and use of computer-based environments for CPS assessment which can allow individuals to demonstrate their capabilities in complex situations akin to real-world problem solving contexts. The use of computer-based environments further support the capture of all actions and discourse as additional sources of evidence of individuals’ capabilities beyond a final product or answer choice (Honey and Hilton 2011; Quellmalz and Pellegrino 2009). While the introduction of computer-based environments for CPS assessment does support capturing evidence of CPS, which would be difficult to capture with traditional types of assessment, these environments can present challenges, including operationalizing targeted CPS skills at the level of granularity of the data captured in the computer environment and identifying individuals’ CPS skills in the large streams of fine-grained log data generated as a result of individuals’ actions and discourse in the environment (Andrews-Todd and Forsyth 2020; Gobert et al. 2012; Kerr et al. 2016).

Such challenges have informed the design of recent CPS assessments in computer environments. The design of these CPS assessments has utilized either human-agent or human-human collaboration. In human-agent collaboration contexts, a human participant interacts with one or more artificial agents whereas in human-human collaboration contexts, a human participant interacts with one or more other human participants. One notable example of a human-agent approach applied to CPS assessment is the PISA 2015 assessment which surveyed the skills and knowledge of 15-year-old students across more than three dozen countries (OECD 2013b). In their assessment design, a human participant collaborated with one, two, or three computer agents as their team members. This kind of design capitalizes on the ability to tightly constrain the environment in terms of the communication that is allowable by the human. The PISA 2015 assessment further constrained the collaboration by only allowing human participants to communicate via a predetermined set of message options which further supported consistency and control over the interactions (Graesser et al. 2017). Specifically, as the human chooses a response, simple production rules can then determine an appropriate response by the artificial agent. Other assessments have similarly constrained communication with predetermined chat message options (Chung et al. 1999; Herborn et al. 2017; Hsieh and O’Neil 2002; Lin et al. 2015; Rosen and Foltz 2014). However, this comes at a cost as the human is not able to freely respond during collaboration. The cost may be necessary for an international assessment such as PISA to capture a reliable score across multitudes of individuals from different backgrounds, cultures and countries.

Other assessments have utilized human-human collaboration (Andrews-Todd and Forsyth 2020; Care and Griffin 2014; Hao et al. 2015; Liu et al. 2015; Sun et al. 2022; Yuan et al. 2019). These kinds of contexts allow individuals the opportunity to solve a problem in a group in a manner that emulates a real-world environment, thus contributing to ecological validity. This type of collaboration is closer to what occurs when people work together in a group to solve a complex problem, as individuals can produce language as they would in a natural setting in human-to-human conversations. Assessments that support human-human collaboration further allow for the full scope of CPS to be measured with detailed measurement of actions and discourse that individuals engage in at a fine-grained level (Andrews-Todd and Forsyth 2020). Importantly, when human-human collaboration is used in unconstrained or open digital environments, a CPS framework is needed that can support capturing CPS behaviors at the fine grain size of data output in such environments. In the current study, we utilize a CPS ontology that supports identifying important features of CPS a priori at multiple grain sizes (described in detail below, in Section 2.4). An ontology provides a theory-driven representation of a construct and their relationships. The CPS ontology provides a comprehensive model of CPS that incorporates behaviors from existing frameworks and components of prior work in relevant areas such as computer-supported collaborative learning, communication, linguistics, and individual problem solving (Clark 1996; Hesse et al. 2015; Liu et al. 2015; Meier et al. 2007; OECD 2013a, 2013b; Roschelle and Teasley 1995). Behaviors associated with such areas are utilized in the CPS ontology to appropriately capture relevant CPS behaviors around communication, teamwork, and problem solving processes that can be identified in open human-human interaction.

One caveat for utilizing human-human collaboration is that it can create a challenge for understanding the skills attributed to each individual, as the conversations may include numerous potential discourse moves. If the goal was simply to measure an individual’s knowledge of a well-defined domain (e.g., mathematics) under discussion, this could easily be interpreted by pattern-matching expected responses. However, the goal is not to measure well-defined domain knowledge but rather the complex and multifaceted aspects of CPS such as sharing information or perspective taking, which can all take the form of a multitude of discourse moves. In many instances, human-driven approaches (e.g., qualitative coding) have been used to interpret individuals’ discourse in CPS contexts. While such approaches typically facilitate valid and reliable interpretations of individuals’ behaviors, these approaches can be resource intensive and time-consuming, particularly for large-scale datasets (Hao et al. 2019). Human annotation requires more than one rater, time to train raters, time to ensure reliability between raters, and time for the raters to view and annotate the large streams of data from computer-based environments. These aspects make human-driven approaches for interpreting CPS data a challenge to implement at scale. Fortunately, there have been advances with machine-driven approaches that can potentially address some of these challenges.

### 1.2. Machine-Driven Approaches for CPS Assessment

Recent research has shown that the interpretation of CPS data can be automated using natural language processing (NLP) approaches (Flor et al. 2016; Flor and Andrews-Todd 2022; Hao et al. 2017; Pugh et al. 2021; 2022; Stewart et al. 2019). As previously noted, one approach for CPS assessment is to have humans interact with an artificial agent to solve a problem. With this type of environment, the natural language about a well-defined domain (e.g., mathematics) can be captured with established methods such as following Expectation-Misconception dialogue which emulates expert tutor moves or responses to student answers (Graesser et al. 2004). In this framework, all NLP is focused on matching the domain-specific answer to an expectation via regular expressions (Jurafsky and Martin 2008) and Latent Semantic Analysis (LSA) (Landauer et al. 2007). Regular expressions focus on capturing a word or phrase whereas LSA is a geometric pattern matching algorithm to determine similarity between the human input within the context of the environment to statements in a larger corpus. Regular expressions have been quite useful in determining an expected human response, comparable to expert human raters (Cai et al. 2011). Among other studies, LSA has been well applied to measure essay quality (for a review, see Landauer et al. 2007) and similarity in contributions among group members (Dowell et al. 2019). Although these computational approaches work well for well-defined domains and general writing quality, neither of these approaches may adequately capture the complexity of fine-grained measures associated with CPS.

In other work, researchers have attempted sentiment analysis (Hao et al. 2019) and generating student profiles based on various NLP metrics (Dowell et al. 2019; Dowell et al. 2020). Indeed, sentiment analysis may uncover some of the intent behind language; however, it may not entirely capture specific aspects of CPS (e.g., identifying instances of perspective taking in fine-grained log data). Profiles of students based on responsivity, given-new metrics and other staples of NLP have been a solid start and replicated across domains. These profiles provide types of collaborators (e.g., Drivers, Influential Actors) based on certain kinds of behaviors (Dowell et al. 2020). This approach is quite useful for determining problem-solver types to characterize individuals or use for optimal groupings but does not provide the detailed information necessary with respect to specific low-level CPS skills.

Additional NLP work has sought to provide detailed information for specific low-level CPS skills. In this work, data of communication among team members are analyzed by NLP algorithms that have been trained to identify CPS behaviors by human-annotated datasets. For example, one group of researchers developed an automated annotation system called CPS-rater which automatically labels communication data according to a specified CPS rubric or framework. This system takes into account the interdependency among participant turns in communication data to improve accuracy (Hao et al. 2017). Other work has similarly utilized models that take into account adjacent utterances (Pugh et al. 2021). Specifically, this work has applied a deep transfer learning approach using the Bidirectional Encoder Representations from the Transformers (BERT) model with a special input representation that considers previous and subsequent utterances for contextual cues. These kinds of algorithms can outperform other developed algorithms for identifying collaborative behaviors that treat each individuals’ turn as independent utterances (Flor et al. 2016; Rosé et al. 2008). Further work has used Random Forest classifiers that use n-grams features (counts of words or phrases) or features derived from the Linguistic Inquiry and Word Count (LIWC) dictionary (word categories from the dictionary) to identify CPS behaviors (Pugh et al. 2022; Stewart et al. 2019). Research has shown BERT and models based on LIWC generated features can have good accuracy and generalizability across task contexts, with BERT models potentially better suited for large datasets and a LIWC approach better suited for small datasets (Pugh et al. 2022).

### 1.3. The Current Study

In much of the current CPS assessment work that aims to identify CPS skills from open dialogue, human-driven approaches (e.g., qualitative coding) are used to identify individuals’ CPS skills. Recent advances in machine-driven approaches offer promise in providing a means to circumvent the time- and labor-intensive human approaches to automatically detect CPS skills. In moving to automatically identifying CPS skills from individuals’ communications and actions, less attention has been given to the reliability or comparability of such approaches compared with human-driven approaches when using themto make inferences about individuals’ CPS skills (Flor et al. 2016; Hao et al. 2017; Pugh et al. 2021; Stewart et al. 2019). In the current study, we aim to compare CPS skill profiles that utilize human annotation to identify individuals’ CPS skills to profiles that use automated annotation to identify individuals’ CPS skills. To extend prior work that utilized a dimensional profile approach (Andrews-Todd and Forsyth 2020), in the current study, we use a typological profile approach. A typological approach affords cutting across both CPS dimensions to characterize individuals’ behaviors. We seek to explore how well the approaches align in terms of generating the same profiles and how comparable student classifications into identified profiles are across the annotation approaches. Based on recent work (Flor and Andrews-Todd 2022) and the extent to which automated approaches are becoming more sophisticated, our expectation was that the same profiles would emerge across both annotation methods. However, given that automated approaches are not typically 100% accurate in identifying CPS skills, we believed there would be differences in how students were categorized into profiles across the two annotation approaches. Our research questions are listed below.

RQ1: Do the same profiles emerge from data derived from human annotation and automated annotation?

RQ2: To what extent are student participants categorized according to the same profiles across data derived from human annotation and automated annotation?

## 2. Materials and Methods

### 2.1. Participants

The study included 378 participants who completed the study in groups of three (i.e., 126 groups). The study participants came from 26 United States postsecondary institutions, recruited through a multi-faceted approach (Steinberg et al. 2020). For participant gender, 76% of participants identified as males, 21% identified as female, and 3% of participants either reported ‘Other”, preferred not to respond, or were unreported. For gender composition of the groups, 56% of the groups (70 of 126 groups) were of the same gender and 44% (56 of 126 groups) were of mixed gender. For participants’ race/ethnicity, 62% of participants identified as White, 7% identified as Black or African American, 8% identified as Asian, 10% reported being more than one race, 1% reported ‘Other’, and 4% preferred not to respond or were unreported. Seven percent of students identified as Hispanic. Participants ranged in age from 18 to 35 years old, with the most frequently reported age range being 18–20 years old. Years of schooling among student participants ranged from 11 to 20 years, with most reporting having 14 years of schooling (i.e., sophomore).

According to the Integrated Postsecondary Education Data System (2019), the sample represented institutions from all four primary geographic regions in the United States, and consisted of both two-year and four-year institutions, including seven minority-serving institutions (MSIs), serving a variety of undergraduate student population sizes. The participating four-year institutions represented a range of undergraduate selectivity (Barron’s Educational Series 2017). There were 32 different class instructors and the proportion of students varied by content area (electronics = 14%; engineering = 12%; general science = 60%; cross-domain = 14%) and difficulty level (beginner = 63%; intermediate = 31%; other = 6%).

### 2.2. Task

**Figure 1:**
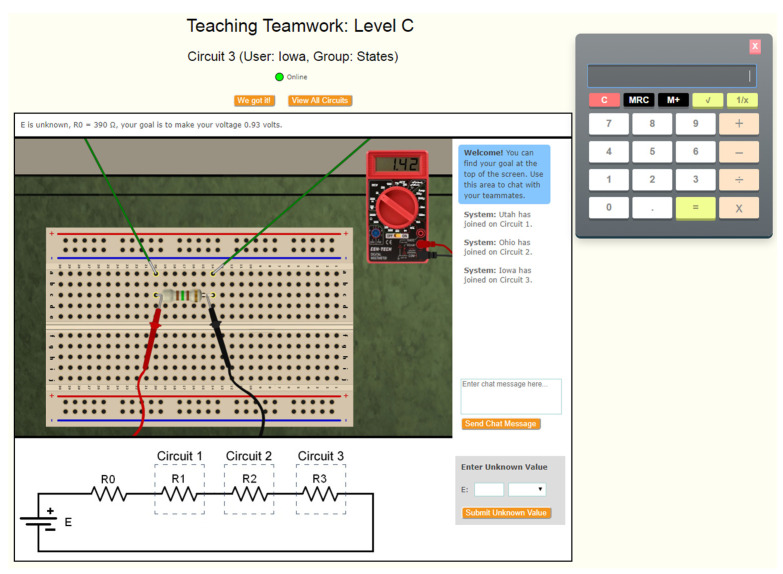
Screenshot of the Three-Resistor Activity interface.

The task that students completed was called the Three-Resistor Activity (Horwitz et al. 2017). Students completed the task in groups of three, with each student working on a separate computer that ran a simulation of an electronics circuit. Each group member’s simulation connected to form a series circuit. The task interface included a calculator, a chat window to communicate with teammates, a digital multimeter (DMM) with two probes to take measurements, a resistor to make resistance changes for the circuit, a zoom button (View All Circuits) to view the circuit boards of other team members, and a submit button (We got it!) to submit answer choices. Figure 1 provides a screenshot of the task interface.

When completing the task, students had the goal of reaching a specified goal voltage value on each of their circuit boards. Since each team members’ circuits were connected in series, any change made by one team member would affect the readings on each team members’ circuit board. Thus, students needed to communicate with each other to coordinate their actions so that each student could reach their goal voltage value. There were four levels in the task that increased in difficulty, with each subsequent level presenting a more difficult problem or a reduction the amount of information provided to students. Table 1 provides an overview of the characteristics of each task level. Students had approximately one hour to complete the task during a class session.

### 2.3. Measures

Prior to completing the Three-Resistor Activity, students completed a series of pre-surveys, including a background information questionnaire (e.g., age, gender, race, language, year in school, mothers’ education level, and preferences for working alone) and a content knowledge pre-test to evaluate students’ electronics content knowledge. The content knowledge pre-test consisted of 23 items across three areas: properties of series circuit, knowledge of electrical laws, and properties of digital multimeters (Steinberg et al. 2020). After completing the Three-Resistor Activity, students completed a series of post-surveys, including a post-task experience survey (with questions related to how much effort students put into the activity or what kinds of difficulties students experienced as examples) and a CPS Inventory in which students provided a self-reported rating on the extent to which they displayed social CPS behaviors (e.g., “I shared information that helped to solve the problem”) and cognitive CPS behaviors (e.g., “I helped to develop a plan to solve the problem”). Students also completed a Team CPS Inventory in which they rated the extent to which their team as a whole displayed social and cognitive CPS behaviors.

### 2.4. CPS Ontology (Framework)

We used a CPS ontology to conceptualize the CPS construct. In the context of our work, the CPS ontology provides a representation of the CPS skills, their relationships to each other, and links the skills to observable behaviors in the Three-Resistor Activity that would provide evidence of each skill (Andrews-Todd and Kerr 2019). The top portion of the ontology provides a generalizable construct definition of CPS (e.g., sharing information), completed through an extensive review of prior frameworks and relevant areas of research (e.g., individual problem solving, linguistics, computer-supported collaborative learning) (Clark 1996; Hesse et al. 2015; Liu et al. 2015; Meier et al. 2007; OECD 2013a, 2013b; O’Neil et al. 1995). Each subsequent layer of the ontology introduces more specificity in describing CPS in the context of a particular domain (e.g., sharing status updates) and then within a particular task environment within that domain (e.g., sharing the status of resistance values in the circuit). The links between each of these layers describe how low-level behaviors from a task environment can be aggregated to make inferences about higher level capabilities. As another feature, the CPS ontology lays out the low-level features corresponding to individuals’ social and cognitive CPS behaviors that need to be extracted from the task log files (Andrews-Todd and Forsyth 2020).

The CPS ontology includes nine high-level skills, four of which are included in the social dimension (teamwork, collaboration) and five of which are included in the cognitive dimension (problem solving, task work). The four skills in the social dimension are maintaining communication, sharing information, establishing shared understanding, and negotiating. The five skills in the cognitive dimension are exploring and understanding, representing and formulating, planning, executing, and monitoring.

Maintaining communication corresponds to social, content-irrelevant communication (Lipponen 2000; Lipponen et al. 2003; Liu et al. 2015). Maintaining communication includes three sub-skills, rapport building communication (e.g., greeting teammates, praising teammates), off-topic communication (e.g., discussing what one had for breakfast), and inappropriate communication (e.g., denigrating teammates). Sharing information corresponds to content-relevant information shared in the service of solving the problem (Mesmer-Magnus and DeChurch 2009; Stasser and Titus 1985; van Boxtel et al. 2000; Webb 1991). This includes sub-skills associated with sharing one’s own information (e.g., sharing answer choices, sharing goal voltage values on one’s own board), sharing task or resource information (e.g., sharing the location of the calculator or the Zoom button in the task interface), and sharing the state of one’s understanding (e.g., metacognitive statements such as “I don’t get it”). Establishing shared understanding refers to communication used to learn the perspective of others and ensure that what has been said is understood. This CPS behaviors has roots in the linguistics and communication literature (Clark 1996; Clark and Brennan 1991). Establishing shared understanding includes sub-skills associated with a presentation phase in grounding communication (e.g., requesting information from teammates (“what is your goal voltage?”)) and an acceptance phase in grounding communication (e.g., providing responses to teammates that indicate comprehension of a statement or lack of comprehension of a statement (“I hear you” or requests for clarification)). Negotiating corresponds to communication used to determine if conflicts exist and resolve conflicts when they arise (Brodt and Thompson 2001; Hesse et al. 2015; Kirschner et al. 2009). Negotiating includes the sub-skills expressing agreement (e.g., “you are right”), expressing disagreement (“that’s not right”), and resolving conflicts (Andrews-Todd et al. 2018; Andrews-Todd and Forsyth 2020).

For the cognitive dimension, exploring and understanding corresponds to actions and communication used to build a mental representation of the various individual components of the problem (Frensch and Funke 1995; OECD 2013a). This includes sub-skills for exploring the task environment (e.g., spinning the digital multimeter dial) and trying to understand the problem (e.g., reading instructions quietly to self). Representing and formulating corresponds to communication used to generate a mental representation of the whole problem space (Mayer and Wittrock 1996; OECD 2013a; VanLehn 1996). This includes sub-skills associated with representing the problem (e.g., “this is a series circuit”) and formulating hypotheses (e.g., “I think if everyone has 470 ohms it will be 3.25”). Planning refers to communication used in the service of developing a plan to solve the problem (Cohen 1989; Hesse et al. 2015; OECD 2013a; Wirth and Klieme 2003). This includes sub-skills for setting goals (e.g., “we need to get the number on the red thingy to the number we are assigned”), developing and revising strategies for solving the problem (e.g., “Let’s calculate E first using Kirchhoff’s voltage law”), and managing resources available (e.g., determining who will do what in the team). Executing corresponds to actions and communication used to support carrying out the plan (OECD 2013a; Wirth and Klieme 2003). This includes sub-skills for the actions taken to carry out the plan (e.g., changing the resistor), making suggestions for actions teammates should take to carry out the plan (e.g., “Adjust yours to 300 ohms”), and reporting to or informing others, of what you are doing to enact strategies for solving the problem (e.g., “I’m going to set mine higher”). Monitoring includes actions and communication used to monitor progress towards the goal and monitor team organization (OECD 2013a; 2013b; O’Neil 1999). This includes sub-skills for actions and communication used to monitor the team’s progress in reaching the goal (e.g., clicking the Submit button on the interface to get feedback about success in solving the problem or saying “We got it” or “I got my goal voltage”) and actions and communication to monitor teammates to determine if they are present and following roles or rules of engagement set by the team (e.g., “Where’s [teammate’s name]?” “Let’s get a move on [teammate’s name]” or clicking the Zoom button to see the status of other teammates’ boards). Table 2 provides an overview of the CPS dimensions, skills, and sub-skills. For a more detailed description of the CPS ontology, see Andrews-Todd et al. (2018), Andrews-Todd and Kerr (2019), and Andrews-Todd and Forsyth (2020).

## 3. Analyses

### 3.1. Human Annotation

Three trained human raters coded each Three-Resistor Activity log file event that corresponded to a student generated action (e.g., resistor change) or chat message (50,817 events) for the presence of one of twenty-three CPS sub-skills (which were later aggregated to the nine high-level CPS skills described in the previous section). The CPS skills in our framework include both student actions and communications because both behaviors can provide important evidence for how individuals are interacting in a problem solving context with teammates and the degree of involvement in the problem solving process. Looking at communication behaviors alone can potentially exclude valuable evidence from action-based behaviors that contribute to the group problem solving process. Paying attention to action-based behaviors can also provide information related the extent to which individuals are being cooperative with their teammates (e.g., when a teammate suggests a resistance value for teammates to set and one teammate chooses not to follow the suggestion).

In training the raters, the first author established several training meetings in which tasks included reviewing and learning the CPS rubric and engaging in coding practice rounds as a group and then individually with small sets of task log data. During these practice rounds, the coding team discussed discrepancies in coding to resolve differences of opinion and refine the rubric where needed. To establish inter-rater reliability, the raters then coded 20% of the data (Fleiss Kappa = 0.937, indicating almost perfect agreement (Landis and Koch 1977)). For the reliability stage, all three raters received the same data to code and the same timeline for completing this coding independently. After sufficient inter-rater reliability was achieved, the remaining data were divided among the three raters and coded independently. In this stage of coding, each rater then had different sets of data to code, but the same timeline to complete the coding. After coding was completed, all discrepancies among the raters were resolved through discussion to reach consensus on the final codes. For analyses, two skills that can be displayed as both actions and chats (i.e., executing and monitoring) were divided into separate CPS behaviors (i.e., executing actions, executing chats, monitoring actions, monitoring chats). Thus, there were 11 CPS skills used in subsequent analyses.

### 3.2. Automated Annotation

Automated classification of the data was approached as a hybrid, rule-based and machine-learning classification process (Flor and Andrews-Todd 2022). All the events in the Three Resistor Activity were automatically logged, with multiple information fields. One of the fields describes the recorded type of the event, whether it was a chat message, interaction with the (virtual) equipment, or submitting task results. Separation of chat and non-chat events is easily obtained from such information. Then, we used machine learning for chat messages and a rule-based approach for non-chat events.

For chat messages, we applied a k-nearest-neighbors (kNN) classifier (Cover and Hart 1967). With kNN, a new instance given for classification receives the same label as the majority of its nearest neighbors (most similar cases) for which labels are known. We used semantic similarity between chat messages as the pivot of our approach. Semantic similarity was computed using dense real-valued vectors, known as word embeddings (for a review, see Lenci 2018). The use of embeddings allows for easily bridging across chat messages that have the same words but with different inflections or have synonyms and otherwise semantically related words. For our experiments, we utilized the fastText 300-dimensional word embeddings, which were trained in English Wikipedia (Bojanowski et al. 2017).

We applied several pre-processing steps to the chat texts. All texts were tokenized and converted to lower case. Alphanumeric tokens that were a concatenation of numbers and characters were automatically split into a number part and word part (e.g., *80 ohms* → *80 ohms*). If the chat text contained any numeric tokens (integers or decimals), each such number was replaced by the string “number” (since the exact value of any number was not important for our task). Addtionally, of note is that in the Three-Resistor Activity, participants were required to use ad-hoc player names if they wanted to address each other (e.g., participants in the team *Animals* were given the code-names *Bear*, *Tiger* and *Lion*, see also the illustration in Figure 1). During automated text pre-processing, if such player names were encountered in the chat text of the respective team, they were replaced with the string “person”. In addition, we applied spelling correction and slang normalization. All chat texts were automatically spell-corrected using a modified version of the ETS spell checker (Flor 2012). The spellchecker also normalized and expanded slang words and expressions (e.g., *yeah* → *yes; idk* → *I don’t know*), using a dictionary of slang terms. Punctuation was ignored. After normalizing a text message, embedding vectors for each word were retrieved from a pre-trained language model.

A single vector representation for each chat message was obtained by averaging the vectors of the component words. Tokens for which no embedding was available were omitted from consideration. During the training phase with our data, we computed an average vector for each chat message (all vectors were normalized with L2 normalization). During the classification stage, when a new chat message is classified, we seek *k* most similar to other messages from the training data (human-annotated with CPS labels), where similarity is computed as the cosine measure between the average vectors of the chat messages. The final label is voted by a weighted majority of nearest neighbors.

The accuracy of our kNN classifier was evaluated using a leave-one-out approach. At each step, all available chat messages are used as training data except one, on which the classification is performed. This is repeated for each chat message. This classifier achieved accuracy of 0.715 relative to human-annotated labels of all chat messages. To adjust for chance agreement, we computed the Cohen’s Kappa. The algorithm achieves the Kappa value of k = 0.628, which is within the range of substantial agreement (Landis and Koch 1977).

Classification of non-chat-events proceeded using information from the task logs. Zooming and viewing the virtual boards and click-submitting the results were classified as ‘Monitoring Actions’ (code CM). Using the calculator was categorized as ‘Executing Actions’ (code CE). However, labeling activities for changing the resistor values was nuanced. When human annotators classified such events, they made a distinction about the presumed state of the task participants. If task participants were in ‘exploration mode’, changing resistor values was considered as ‘Exploring and Understanding’ (code CEU). However, as soon as the task participants formulated some kind of a plan of action, changing resistor values was labeled as ‘Executing Actions’ (code CE). Thus, the decision of whether a specific resistor change action is a CE or a CEU event depended on the annotator’s estimation of whether the team is still exploring or already has a plan. Using a calculator can be an indication of formulating a plan, and other indicators can be gleaned from the content of the adjacent chat messages. When participants switched to a new level in their task, annotators considered it as a reset to ‘exploration mode’, until evidence for a new plan was encountered.

For the automated classification of action events, we used a rule-based approach that relied on the recorded action type. For the CE/CEU distinction, the rule-based approach was enriched with the following: on which level the team is working, whether any team member used a calculator in the current level, and whether the automated chat-message-classifier already detected a Planning Chat Event (code CP) among the events of the current level. This is an uncommon case where a rule-based classification of activity events partially depends on the statistical classification of preceding chat messages. Overall, the rule-based classifier achieved accuracy of 0.880 over the set of all non-chat events.

The combined classifier (rule based + machine learning) achieved accuracy of 0.826 over the full dataset (all events), with Kappa value k = 0.765. Table 2 provides a direct comparison of the human and automated annotation methods in terms of the counts and proportions of each CPS skill classified by each annotation method, with representative examples for each CPS skill. It should be noted that, as expected, the successful classification of chats is more difficult than the classification of actions. First, actions were classified into just 3 classes, whereas chats had 8 possible labels. Moreover, the classification of actions relies on less ambiguous information—the only ambiguity is contextual (i.e., in what context some actions appear), whereas chat data are much more ambiguous with respect to CPS labels. However, one of the largest discrepancies was in classification of CEU actions (Exploring and Understanding), with 5309 actions in human annotation, but only 2441 such actions in automated annotation. In a complementary manner, manual annotation counted 23,582 CE actions, while automated annotation assigned the CE label to 26,455 actions. The CE/CEU classification is exactly the case where our rule-based algorithm depended on contextual interpretation (is the team already in a planning stage?), and this is the case that will require particular improvement in future development.

Correlations between displayed CPS skill frequencies across annotation methods were computed as an additional measure of consistency (see Table 3). The frequencies of almost all CPS skills between annotation methods were quite highly correlated (r ≥ 0.83), but exploring and understanding and representing and formulating were far below that (0.59 and 0.53, respectively). While counts of social CPS skills tended to be reasonably inter-correlated across methods, the same cannot necessarily be said for cognitive CPS skills; in fact, some correlated more highly with social skills (e.g., executing chats, monitoring chats, and planning).

### 3.3. ClusterAnalysis

Hierarchical cluster analyses were performed separately on the aggregate frequencies of each CPS code for each person based on the human annotation and the automated annotation following a process described in Forsyth et al. (2020), whereby hierarchical cluster analyses (Ward 1963) were conducted directly on the frequencies of CPS skills exhibited by each participant. We allowed the clusters to emerge from the data without imposing pre-existing theoretical solutions a priori. The final number of resulting profiles was determined based on optimizing the minimum number of subjects in each cluster (n = 20) for validation analyses (see next section). We named the clusters according to how well each profile meaningfully related to constructs and findings from previous research in areas such as social psychology, cognitive psychology, and communication (e.g., Latané et al. 1979; Clark 1996; Stasser et al. 2000). So it was not until the clusters emerged that we sought to name or characterize them based on theory and/or prior research. The characteristics of each profile were defined by standardizing the mean frequency for each skill within each cluster relative to the frequency for the overall sample based on the human annotation or automated annotation. To determine if the profiles from human annotation and automated annotation were the same, we examined the similarities in patterns of standardized frequencies for each skill across each cluster for both annotation methods.

### 3.4. Validation Analyses

The aggregate statistics from the discovered profiles were compared according to their task performance as identified by the number of task levels attempted, performance on the electronics pre-test, and ratings on a post-task self and team CPS Inventory with nonparametric Kruskal-Wallis tests to test for differences across profiles given somewhat limited sample sizes and possible concerns about the underlying normality of the skill frequency distributions within and across clusters given the data shown in Table 2. Monte Carlo simulations were included with these post-hoc tests with Bonferroni corrections for multiple comparisons to ensure accurate statistical significance. Presuming that the profiles would be comparable across annotation methods, the respective cluster solutions were compared on the proportion of cases consistently placed in similar profiles. Correlations between displayed CPS skill frequencies were computed as an additional measure of consistency.

## 4. Results

### 4.1. Human Annotation Cluster Analysis

Consistent with previous research (Andrews-Todd et al. 2018; Andrews-Todd and Forsyth 2020) though with slight differences in proportions, four distinct profiles emerged from the human annotation (see Table 4). The profiles included what we called Social Loafers, Super Socials, Low Collaborators, and Active Collaborators. On the whole, Social Loafers (n = 224; 59.3%) tended to display fewer CPS skills relative to other clusters. Specifically, Social Loafers tended to exhibit CPS skills from approximately 0.3 to 0.4 SDs below the average for the entire sample. Super Socials (n = 99; 26.2%) tended to display higher frequencies of social relative to the cognitive CPS skills. For example, Super Socials displayed far above average use of negotiating (z = 0.89), establishing shared understanding (z = 0.66), and sharing information (z = 0.60), yet they were slightly below average for the sample in exploring and understanding (z = −0.37) and executing actions (z = −0.22). Low Collaborators (n = 21; 5.6%) tended to engage in independent action-based cognitive CPS behaviors more than other clusters, but engaged communicatively very little with teammates. In essence, these students seemed to have attempted to work alone without communicating much with their teammates. This profile was much above average on executing actions (z = 3.02) and exploring and understanding (z = 0.63) relative to other clusters and below average on all communication-based CPS skills (e.g., executing chats (z = −0.43), sharing information (z = −0.66), establishing shared understanding (z = −0.70), and negotiating (z = −0.60). On the whole, Active Collaborators (n = 34; 9.0%) tended to display more CPS skills relative to other clusters. Specifically, they were above average (z ≥ 0.22) compared to the total sample on every CPS skill except monitoring actions which was slightly below average (z = −0.10).

### 4.2. Human Annotation Validation Analyses

There was consistent differentiation across clusters on external measures. There was a significant relationship between cluster membership and task performance, here operationalized as the number of task levels attempted (*X*^2^ (3,370) = 16.90, *p* = 0.001; *partial η*^2^ = 0.05). Super Socials and Active Collaborators attempted the most levels (mean ranks = 207.32 and 205.90, respectively), followed by Low Collaborators (198.50) and then Social Loafers (171.15). Post-hoc analysis showed Social Loafers were significantly lower compared to Active Collaborators (*p* = 0.003) and Super Socials (*p* = 0.03).

Cluster membership was also associated with performance on the electronics pre-test (*X*^2^ (3,370) = 21.55, *p* < 0.001; *partial η*^2^ = 0.06). Super Socials displayed the highest mean ranks (220.90), followed by Active Collaborators (212.31), Social Loafers (170.20), and Low Collaborators (137.45). Post-hoc analysis showed Super Socials significantly outperformed Social Loafers (*p* = 0.001) and Low Collaborators (*p* = 0.007).

On the mean self-ratings from the CPS Inventory, there was a relationship with cluster membership (*X*^2^ (3,370) = 19.87, *p* < 0.001; *partial η*^2^ = 0.05). Super Socials had the highest mean rank (213.25) and had significantly higher ratings than Social Loafers (157.80; *p* < 0.001). Nonetheless, Super Socials still had higher ratings than Low Collaborators (163.82) and Active Collaborators (176.97), though the results were not significant (*ps* > 0.05).

Finally, with respect to mean team ratings from the CPS Inventory, there was also a relationship with cluster membership (*X*^2^ (3,370) = 11.62, *p* = 0.007; *partial η*^2^ = 0.03). A generally similar pattern was discovered as with the self-ratings where mean ranks indicated that Super Socials (203.73) reported the highest ratings for their team and reported significantly higher ratings than Social Loafers (162.42; *p* = 0.006). Super Socials also reported higher ratings on average than Low Collaborators (157.50) and Active Collaborators (180.08), though the results were not significant (*ps* > 0.05).

### 4.3. Automated Annotation Cluster Analysis

The same profiles emerged from the skill profiles derived from automated annotation, with noticeable differences in the proportion of the sample in the Super Socials and Low Collaborators groups, relative to the profiles derived from human annotation (see Table 5). This was perhaps expected given the relative distributions of CPS skills across methods were not presumed to be consistent.

Similar to human annotation, for automated annotation, the Social Loafers (n = 192; 50.8%) tended to exhibit all CPS skills at levels slightly below the average for the entire sample. Relative to the entire sample, the Super Socials (n = 64; 16.9%) displayed above average use of social relative to cognitive CPS behaviors. For example, they showed above average use of negotiating (z = 1.14), establishing shared understanding (z = 1.09), sharing information (z = 0.63), and unlike with the human annotation, maintaining communication (z = 0.60), yet were still slightly below average for the sample in exploring and understanding (z = −0.41) and executing actions (z = −0.20). The profile for Low Collaborators (n = 99; 26.2%) revealed slightly below average demonstration of all communication-based CPS skills but much above average demonstration of action-based CPS skills, including monitoring actions (z = 0.87), executing actions (z = 0.68) and exploring and understanding (z = 0.65). Finally, Active Collaborators (n = 23; 6.1%) were above average (z ≥ 0.22) compared to the total sample on every CPS skill except monitoring actions which was slightly below average (z = −0.27).

### 4.4. Automated Annotation Validation Analyses

As with the human annotation, there was consistent differentiation across clusters on external measures with the automated annotation. There was a significant relationship between cluster membership and task levels attempted (*X*^2^ (3,370) = 13.10, *p* = 0.004; *partial η*^2^ = 0.04). Active Collaborators and Super Socials attempted the most levels (mean ranks = 221.37 and 208.93, respectively), followed by Low Collaborators (188.06) and then Social Loafers (172.04). Post-hoc analysis showed Social Loafers were significantly lower performers compared to Super Socials (*p* = 0.02).

Cluster membership was associated with performance on the electronics pre-test (*X*^2^ (3,370) = 15.34, *p* = 0.003; *partial η*^2^ = 0.04). Active Collaborators displayed the highest mean ranks (233.72), followed by Super Socials (215.99), Social Loafers (179.14), and Low Collaborators (166.26). Post-hoc analysis showed Super Socials and Active Collaborators significantly outperformed Low Collaborators (*p*s = 0.025 and 0.039, respectively).

On the mean self-ratings from the CPS Inventory, there was a relationship with cluster membership (*X*^2^ (3,370) = 22.64, *p* < 0.001; *partial η*^2^ = 0.07). Super Socials had the highest mean rank (223.50) and had significantly higher ratings than Low Collaborators (148.94; *p* < 0.001) and Social Loafers (167.73; *p* = 0.001). Super Socials also had higher ratings than the Active Collaborators (198.52), but this difference was not significant (*p* > 0.05).

Finally, with respect to mean team ratings from the CPS Inventory, there was also a relationship with cluster membership (*X*^2^ (3,370) = 15.31, *p* < 0.001; *partial η*^2^ = 0.04). A generally similar pattern was discovered as with the self-ratings where Super Socials (216.25) showed the highest ratings and had significantly higher ratings than Low Collaborators (156.08; *p* = 0.002) and Social Loafers (167.60; *p* = 0.006). Super Socials had higher ratings on average than Active Collaborators (191.75) as well, but the difference was not significant (*p* > 0.05).

### 4.5. Comparing Clusters across Annotation Methods

**Figure 2:**
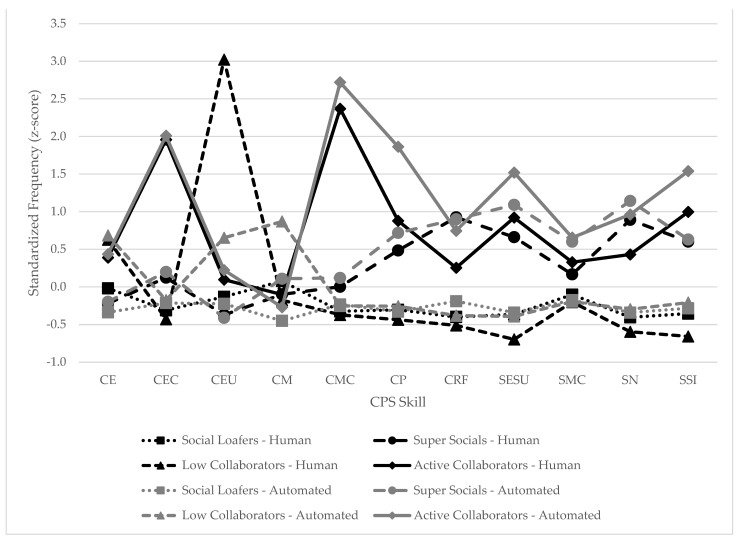
Line plot of standardized frequencies of CPS skills by cluster and annotation method.

Presuming that the profiles would be reasonably comparable across annotation methods, we calculated the Spearman rank correlation of the frequencies for each CPS skill for each cluster between annotation methods. Results in fact showed reasonably high similarity between methods (*r_s_* range = 0.83–0.89; *p* < 0.002). A line plot of the standardized frequencies of each CPS skill for each cluster across annotation methods can be found in Figure 2. The respective cluster solutions were compared on the proportion of cases consistently placed in similar profiles (see Table 6 for a comparison of cluster solutions across annotation methods). The results showed that 62.4% (n = 226) of participants were placed in the same cluster between the human and automated annotation methods. When controlling for the human annotation cluster assignments, the respective consistency rates were 65.6% (Social Loafers; 147/224), 55.6% (Super Socials; 55/99), 71.4% (Low Collaborators, 15/21), and 55.9% (Active Collaborators, 19/34). Additionally, the Bowker (1948) generalization of the McNemar test for symmetry produced a significant result (*X*^2^ (6,378) = 94.55, *p* < 0.001, meaning there was a difference in the cluster assignments between methods.

## 5. Discussion

Collaborative problem solving is critical for many educational and workplace contexts and this has led to numerous research endeavors aimed at developing approaches for measuring and developing CPS skills. Some of the prior research has utilized constrained designs that limit collaboration (e.g., with human-agent collaboration and/or predetermined message options for communication) and constrain the problem space to support standardization of assessments (Hao et al. 2015; Herborn et al. 2017; Lin et al. 2015; OECD 2013b), but these design decisions do not allow for the full scope of CPS to be measured. Allowing open communication among human participants in open-ended digital environments make for situations that more closely align to real-world collaborative situations that individuals may encounter in their everyday lives, thus supporting ecological validity. In CPS work that has utilized such contexts, most have used human annotation to identify evidence of CPS skills in the data. Such annotation methods can be time-consuming and resource intensive. Machine-driven approaches that support automated annotation can help circumvent this issue, but the use of these approaches in the context of CPS assessment is in its infancy.

In the current study, we annotated data from an open online collaborative simulation-based task on electronics concepts using trained humans and automated methods. We sought to compare CPS skill profiles that emerged from the data derived from the two annotation methods in an effort to determine the extent to which the two annotation methods align. We further sought to explore the extent to which students were similarly categorized with respect to the CPS skill profiles across the two annotation methods. In line with our hypothesis, the same four CPS skill profiles emerged across the data derived from the human and automated annotation. We named these profiles Social Loafers, Super Socials, Low Collaborators, and Active Collaborators. Social Loafers were students who tended to sit back and not contribute much in terms of any of the CPS skills during task engagement in comparison to other profiles. Interestingly, across both annotation methods, this profile occurred most frequently. Individuals in this profile could also be called “Free Riders” (N. L. Kerr and Bruun 1983), as they appeared to reduce their effort and allow other teammates to carry more of the load. The labeling of individuals according to these names are made in light of theoretical interpretations of how their behaviors align with social psychological theory associated with the constructs (Latané et al. 1979; N. L. Kerr and Bruun 1983). However, it is worth noting that this behavior of social loafing or free riding can be situational and should not be interpreted as a trait of an individual, as individuals can behave differently in another situation. Super Socials contributed a great deal of social CPS skills (e.g., sharing information, negotiating) relative to cognitive CPS behaviors, particularly those that were action-based skills (e.g., executing actions, exploring and understanding). Low Collaborators tended to be less inclined to collaborate with their partners, instead working independently using individual action-based CPS skills (i.e., executing actions (e.g., changing the resistor, performing calculations), and exploring and understanding). Active Collaborators displayed most CPS skills frequently in comparison to other profiles, thus demonstrating a high level of active participation throughout the CPS process.

The patterns of the profiles are consistent with results from prior CPS assessment work that has explored profiles that emerge based on students’ participation in computer-based collaborative tasks (Andrews-Todd et al. 2018; Forsyth et al. 2020; Herborn et al. 2017). Specifically, prior work has shown profiles that tend to be on the opposite ends of the spectrum in terms of participation in the CPS process, with a profile the corresponds to less activity relative to others and a profile that corresponds to being very active in the CPS process (Andrews-Todd et al. 2018; Forsyth et al. 2020; Dowell et al. 2020; Herborn et al. 2017). Profiles that have a bit more nuance in terms of CPS behaviors have also been similarly found in prior research. For example, Herborn et al. (2017) designated a profile in their work as “Compensating Collaborators” and they were characterized as having high collaboration actions but performed poorly on problem solving variables. This profile could be usefully compared to Super Socials in the current work.

The profiles that emerged in the current research can provide useful ways to characterize individuals’ CPS behaviors to support assessment and training efforts. For example, after completing a task, a particular stakeholder (e.g., student, teacher, employee, employer) can receive information about the CPS skill profile that characterized a user’s behavior to provide information about the ways in which the individual interacted with teammates and contributed to the CPS process. The information provided by the profiles can also be used to subsequently provide feedback that can address weaknesses noted as part of the profile (e.g., Low Collaborators could be provided feedback to engage in more communicative participation with teammates so that their voices are heard and contributions are acknowledged). It is worth noting that the current profile method for characterizing individuals’ CPS behaviors applies to an individual’s behavior in that specific context (i.e., at that time, in that task, with that team) so it is not necessarily stable over time. It is entirely possible that when an individual engages with a different task or a different team, the CPS behaviors may change and they may demonstrate behaviors associated with another profile. This could be because the individual has more or less interest or prior knowledge with the task or domain or because they are paired with teammates with a different set of personalities. Recent work has explored stability of profiles or CPS skills across tasks and found mixed results, with one showing evidence for generalizability of negotiation skills across tasks (Martin-Raugh et al. 2020) and another showing fewer than half of participants (37%) showing behaviors associated with the same CPS skill profile across mathematics and physics tasks (Andrews-Todd et al. 2021). Future work will be needed to continue exploring the extent to which CPS skill profiles may be stable across different contexts, as the generalizability of individuals’ CPS skills across contexts may depend on a number of factors (e.g., task type, task domain, group size, teammate personalities, communication modality) (Andrews-Todd and Forsyth Forthcoming). 

Validation of clusters across annotation methods showed mostly similar patterns of results, though there were some differences. With respect to task performance, Active Collaborators and Super Socials tended to show the highest number of levels attempted, with Active Collaborators performing best in the human annotation and Super Socials performing best in the automated annotation. Further, for human automation, the only significant differences suggested Social Loafers performed worse than Active Collaborators and Super Socials, while for the automated annotation the difference was only between Social Loafers and Super Socials. Prior research has also shown benefits to performance in collaborative situations when individuals show increases in social and cognitive CPS behaviors (Forsyth et al. 2020; Andrews-Todd and Forsyth 2020; Herborn et al. 2017). Furthermore, research has shown particular benefits of social CPS behaviors (e.g., sharing information, negotiation) for performance outcomes (Hao et al. 2019, 2016; Sun et al. 2022), thus supporting the finding for why Super Socials performed just as well as Active Collaborators.

For the content pre-test, similar results were shown across annotation methods, with Super Socials and Active Collaborators performing best on the test; however the significant differences were different with Super Socials significantly outperforming Social Loafers and Low Collaborators in the human annotation, but Super Socials and Active Collaborators significantly outperforming only Low Collaborators in the automated annotation. These results, with Super Socials and Active Collaborators tending to show highest performance on the content pre-test suggests their higher prior knowledge may have influenced their willingness to contribute substantially to the team collaboration and problem solving. The opposite could be possible for the Social Loafers and Low Collaborators who had the lowest average scores on the pre-test. For example, it could be that the Low Collaborators did not have sufficient prior knowledge to contribute substantially to the work or perhaps they were embarrassed by their level of knowledge and so preferred to work alone (Forsyth et al. 2020). Similarly, Social Loafers could have demonstrated behaviors associated with general inactivity relative to other profiles because of their lower prior knowledge in the current task. Interestingly, prior work has suggested that if individuals are unable to contribute to the group because of lack of competence, other teammates may be willing to compensate by increasing their effort to support group goals (Hütter and Diehl 2011; Kerr 1983). As such, in future work it would be interesting and worthwhile to explore group dynamics in terms of group compositions that include different constellations of profiles and investigate how teammates respond to Social Loafers and how group performance may be affected by having teammates of particular profiles in their teams. For example, in the context of the Three-Resistor Activity, prior work has shown that having at least one team member demonstrating both high levels of social CPS skills and cognitive CPS skills was associated with higher task performance relative to teams without such a team member (Andrews-Todd and Forsyth 2020), but how might group dynamics and team performance be affected by having teammates who display different kinds of CPS behaviors?

For self and team ratings of CPS behaviors displayed, Super Socials provided the highest ratings across annotation methods, with those ratings significantly higher than only Social Loafers for human annotation, but significantly higher than both the Social Loafers and Low Collaborators for automated annotation. The patterns of the students’ ratings are in line with what would be expected given the behaviors indicative of each profile. Specifically, Super Socials and Active Collaborators should report higher ratings given they engaged in more CPS behaviors relative to Social Loafers and Low Collaborators. In the current study, we collected CPS ratings at the team level to capture information about teammates’ behaviors, but we also could have asked students to provide ratings for each individual teammates’ CPS skills which could more clearly map behaviors onto the identified profiles. Due to an already time-intensive data collection, we were unable to do this in the current study, but it would make for an interesting contribution to explore in future work the extent to which individuals’ ratings of each teammate on the CPS Inventory align with teammates’ own ratings and actual in-task CPS behaviors.

The differences that emerged for the cluster validation across the annotation methods were likely because although the same clusters were found, in line with our hypothesis, students were not always categorized into the same profiles across annotation methods. Most of the students (62.4%) were categorized according to the same profiles; however, there was a significant difference in cluster assignments across the annotation methods. In particular, some profiles showed more consistency (e.g., Low Collaborators (71.4%)) than others (e.g., Super Socials (55.6%), Active Collaborators (55.9%)). These results are reasonably in line with prior automated annotation work for CPS which has shown a general, but not complete agreement when compared to human annotation, which is often considered the ground truth (Flor and Andrews-Todd 2022; Flor et al. 2016; Hao et al. 2017; Pugh et al. 2021; Rosé et al. 2008; Stewart et al. 2019). Refinement of our automated annotation algorithms is still ongoing, striving for even better agreement with human annotations. For example, in our ongoing work we are exploring additional contextual features that could potentially improve classification accuracy.

### Limitations and Future Work

Like all studies, this study has limitations as well. Though our sample size was large relative to some collaboration research, the sample size did limit our ability to develop more robust clustering algorithms for student profiles (e.g., k-means instead of Ward’s method). In future work with larger datasets, we could explore other clustering approaches (e.g., latent class analysis) to determine what kinds and if similar profiles emerge. Another limitation is that our study only focused on an adult college population. It will be important for future work to explore the current automated approaches with other populations of individuals to determine if similar patterns of results are found. Further, participants in this study only completed one task. Some recent work has explored the comparability of profiles derived from human and semi-automated methods across task domains, with some differences shown across domains (Andrews-Todd et al. 2022). Thus, future work should continue exploration into the comparability and reliability of these annotation methods across different contexts, with work also aimed at finding ways to optimize generalizability across contexts (see Pugh et al. 2022 as an example), validation methods (e.g., using medians rather than means if underlying normality is a concern in evaluating relative skill distributions), and the ability to predict the likelihood any participant may be placed in a particular profile based on a set of demonstrated CPS behaviors.

The task used in the current study also required sufficient content knowledge to solve the problem. One question for future work is whether different kinds of tasks, including those that do not require domain knowledge, yield similar results in terms of the kinds of profiles represented in the current study and the comparability of the annotation methods. This is important for understanding the interplay between domain knowledge and group performance on collaborative tasks since according to (Simoni et al. 2004), high domain knowledge alone does not necessarily ensure success in such situations. By extension, other aspects of group dynamics (e.g., team gender composition) are important to consider as well, for which exploratory research by Steinberg et al. (2018) did show that all male teams outperformed all female teams or teams of mixed genders. Additionally, it is worth investigating consistency of cluster assignment as it has been shown certain individual personas exhibited in collaborative task environments can affect team performance (Eaton et al. 2017). Future studies can also explore the use of different natural language processing or machine learning approaches to automate the identification of individuals’ CPS skills. It is possible that models different from those used in the current study may yield results more comparable to human annotation for these data or data that are like the current data. Additionally, further investigations are warranted with the CPS Inventory for understanding the relationship between the frame of reference for the perceptions (self vs. team) and the types of skills being rated (social dimensions vs. cognitive dimensions).

## 6. Conclusions

Implications from this work include making strides forward in addressing the challenges of assessing CPS. One such challenge is the laborious work of using trained humans to hand code each discourse move provided by an individual participant interacting with other humans to solve a problem at hand. This is especially a challenge when dealing with large scale datasets. As previously noted, natural language processing techniques to identify CPS skills is not as simple as assessing more well-defined domains such as mathematics. Instead, these techniques when used in the context of CPS assessment requires understanding sometimes complex discourse moves between various types of individuals while they solve a problem. Developing and refining automated approaches for CPS assessment is an important and necessary step in removing the costly and time-consuming practice of human annotation. As an example, in the current work, human annotation took a few months for training and coding relative to the 3 min required for running the automated algorithms. The development of such automated approaches further lay the groundwork to be able to analyze individuals’ group communications on the fly which can support both the assessment and development or training of CPS skills. For assessment purposes, the current work provides preliminary evidence that the automated annotation approach can be used to identify individuals’ CPS skills and support reporting about individuals’ CPS behaviors in terms of what kinds of CPS skill profiles they display, though for formative purposes rather than high-stakes purposes. For training purposes, automated approaches like the one shown in the current study can support formative feedback for individuals regarding potential strengths and weaknesses according to what kinds of CPS behaviors are displayed in a given situation or what kinds of profiles they display during a task or set of tasks. Given that CPS assessment and training are becoming a key focus in educational and workplace contexts, the current research provides important groundwork in supporting efforts to scale up CPS assessment in ways that allow the full scope of CPS to be measured in ecologically valid contexts with open communication between humans and open-ended tasks that align with everyday activities in relevant contexts such as school and the workplace.

## Figures and Tables

**Table 1:** Overview of Three-Resistor Activity levels.

Task Level	External Voltage (E)	External Resistance (R0)	Goal Voltages
1	Known by all teammates	Known by all teammates	Same for all teammates
2	Known by all teammates	Known by all teammates	Different for all teammates
3	Unknown by teammates	Known by all teammates	Different for all teammates
4	Unknown by teammates	Unknown by teammates	Different for all teammates

**Table 2:** Counts and proportions of CPS skills identified across annotation methods with exemplars.

Code	CPS Skill	CPS Sub-Skill	Count for Human Annotation	Count for Automated Annotation	Example
	Social Dimensions (chat messages)				
SESU	Establishing Shared Understanding	Presentation Phase in Grounding	3319 (6.5%)	3629 (7.1%)	*“What is your resistance?”*
		Acceptance Phase in Grounding			
SMC	Maintaining Communication	Rapport Building Communication	1328 (2.6%)	938 (1.8%)	*“Good job yall”*
		Off-Topic Communication			
		Inappropriate Communication			
SN	Negotiating	Express Agreement	1153 (2.3%)	942 (1.9%)	*“Actually, no you can’t”*
		Express Disagreement			
		Resolve Conflict			
SSI	Sharing Information	Share Own Information	6182 (12.2%)	6635 (13.1%)	*“My goal is to make my voltage 3.5”*
		Share Task/Resource Information			
		Share Understanding			
	Cognitive Dimensions (chat messages)				
CRF	Representing and Formulating	Represent the Problem	357 (0.7%)	372 (0.7%)	*“Given earlier fiddlings I’ve deduced that we can’t go lower than 20 DCV”*
		Formulate Hypotheses			
CP	Planning	Set Goals	1070 (2.1%)	1223 (2.4%)	*“Use your resistance and your voltage to get the current. We know the formula, so we might be able to get E and work backwards”*
		Manage Resources			
		Develop Strategies			
CMC	Monitoring communication	Monitor Success	1194 (2.3%)	959 (1.9%)	*“Come on Tiger”*
		Monitor Group			
CEC	Executing communication	Suggest/Direct Actions	1350 (2.7%)	1252 (2.5%)	*“Plum move yours to 150 as well”*
		Report Actions			
	Non-Chat Activities				
CM	Monitoring actions	Monitor Success	5973 (11.8%)	5971 (11.8%)	Viewing board; opening zoom; submitting results
		Monitor Group			
CE	Executing actions	Enact Strategies	23,582 (46.4%)	26,455 (52.1%)	Changing resistor value; using calculator
CEU	Exploring and Understanding	Explore the Environment	5309 (10.4%)	2441 (4.8%)	Changing resistor value prior to developing a plan
		Understand the Problem			
Total events			50,817	50,817	

**Table 3:** Correlation of CPS codes across annotation methods.

Measure	1	2	3	4	5	6	7	8	9	10	11
1. Executing actions	0.96 **	0.11 *	0.31 **	0.10 *	0.12 *	−0.02	−0.09	−0.02	−0.02	0.05	0.08
2. Executing chats	0.06	0.88 **	0.07	−0.02	0.51 **	0.46 **	0.22 **	0.52 **	0.19 **	0.25 **	0.51 **
3. Exploring & Understanding	0.45 **	−0.06	0.59 **	−0.02	0.03	−0.15 **	−0.18 **	−0.19 **	−0.04	−0.17 **	−0.12 *
4. Monitoring actions	0.09	−0.05	0.01	1.00 **	−0.05	−0.03	−0.01	−0.04	−0.03	0.01	−0.08
5. Monitoring chats	0.10 *	0.57 **	0.15 **	−0.04	0.90 **	0.34 **	0.14 **	0.50 **	0.15 **	0.23 **	0.45 **
6. Planning	−0.03	0.42 **	−0.08	−0.04	0.36 **	0.83 **	0.37 **	0.48 **	0.19 **	0.28 **	0.42 **
7. Representing and Formulating	−0.09	0.12 *	−0.13 *	−0.03	0.14 **	0.41 **	0.53 **	0.39 **	0.20 **	0.36 **	0.37 **
8. Establish Shared Understanding	−0.06	0.52 **	−0.13 **	−0.04	0.42 **	0.53 **	0.46 **	0.93 **	0.26 **	0.50 **	0.64 **
9. Maintaining Communication	−0.02	0.10	−0.02	−0.01	0.13 **	0.24 **	0.09	0.31 **	0.95 **	0.15 **	0.28 **
10. Negotiating	−0.09	0.28 **	−0.14 **	−0.02	0.23 **	0.40 **	0.44 **	0.55 **	0.19 **	0.86 **	0.44 **
11. Sharing Information	0.03	0.46 **	0.05	−0.08	0.43 **	0.49 **	0.44 **	0.66 **	0.25 **	0.45 **	0.96 **

Note: ** *p* < 0.01; * *p* < 0.05. Lower diagonal elements refer to the human coding; upper diagonal elements refer to the automated coding; diagonal elements in bold are between methods.

**Table 4:** Descriptive statistics for average frequencies of human CPS codes for each cluster and overall.

	Social Loafers (n = 224)	Super Socials (n = 99)	Low Collaborators (n = 21)	Active Collaborators (n = 34)	Total (n = 378)
Code	Mean (SD)	Mean (SD)	Mean (SD)	Mean (SD)	Mean (SD)
Executing actions	61.3 (54.5)	50.4 (33.5)	96.5 (102.8)	83.5 (49.4)	62.4 (54.5)
Executing chats	2.2 (2.4)	4.1 (2.9)	1.7 (3.1)	12.3 (7.9)	3.6 (4.4)
Exploring and Understanding	11.5 (12.8)	6.9 (10.2)	71.4 (18.0)	15.9 (14.9)	14.0 (19.0)
Monitoring actions	17.0 (17.8)	14.3 (11.0)	13.0 (11.7)	14.2 (11.0)	15.8 (15.4)
Monitoring chats	1.9 (2.0)	3.2 (2.1)	1.8 (1.9)	12.1 (5.2)	3.2 (3.8)
Planning	1.7 (2.1)	4.5 (3.8)	1.3 (2.1)	5.9 (6.3)	2.8 (3.5)
Representing and Formulating	0.4 (0.7)	2.2 (1.8)	0.2 (0.4)	1.3 (1.4)	0.9 (1.4)
Establishing Shared Understanding	6.2 (4.1)	13.5 (8.0)	3.8 (3.6)	15.4 (10.1)	8.8 (7.1)
Maintaining Communication	2.7 (9.0)	4.8 (4.2)	2.0 (2.7)	6.0 (7.1)	3.5 (7.7)
Negotiating	1.9 (1.7)	5.7 (3.4)	1.3 (1.3)	4.3 (3.4)	3.1 (3.0)
Sharing Information	12.6 (7.7)	22.7 (8.6)	9.4 (7.1)	26.9 (16.2)	16.4 (10.6)

**Table 5:** Descriptive statistics for average frequencies of automated CPS codes for each cluster and overall.

	Social Loafers (n = 192)	Super Socials (n = 64)	Low Collaborators (n = 99)	Active Collaborators (n = 23)	Total (n = 378)
Code	Mean (SD)	Mean (SD)	Mean (SD)	Mean (SD)	Mean (SD)
Executing actions	50.4 (31.4)	58.4 (39.6)	109.7 (82.4)	95.2 (53.6)	70.0 (58.0)
Executing chats	2.4 (3.0)	4.1 (3.0)	2.6 (2.5)	11.6 (8.4)	3.3 (4.1)
Exploring & Understanding	4.1 (5.6)	2.2 (4.9)	13.2 (15.2)	8.7 (11.9)	6.5 (10.3)
Monitoring actions	8.8 (7.4)	17.5 (12.4)	29.2 (20.2)	11.6 (9.6)	15.8 (15.4)
Monitoring chats	1.8 (2.0)	2.9 (1.9)	1.7 (1.8)	11.0 (4.9)	2.5 (3.1)
Planning	2.1 (2.0)	5.7 (3.1)	2.4 (2.2)	9.5 (6.3)	3.2 (3.4)
Representing and Formulating	0.7 (1.0)	2.2 (1.7)	0.5 (0.6)	2.0 (1.8)	1.0 (1.3)
Establishing Shared Understanding	7.0 (4.5)	17.8 (7.2)	6.7 (4.3)	21.0 (11.4)	9.6 (7.5)
Maintaining Communication	1.7 (2.0)	5.3 (9.0)	1.6 (2.4)	5.5 (6.1)	2.5 (4.6)
Negotiating	1.6 (1.6)	5.4 (3.0)	1.7 (1.8)	5.0 (3.1)	2.5 (2.6)
Sharing Information	14.4 (8.5)	24.6 (9.3)	15.2 (8.4)	34.8 (19.7)	17.6 (11.2)

**Table 6:** Comparison of cluster analysis results based on counts of participants in each cluster across annotation methods.

	Automated Annotation				
Human Annotation	Social Loafers	Super Socials	Low Collaborators	Active Collaborators	Total
Social Loafers	147	5	72	0	224
Super Socials	32	55	8	4	99
Low Collaborators	6	0	15	0	21
Active Collaborators	7	4	4	19	34
Total	192	64	99	23	378

## Data Availability

The data presented in this study are available for free upon request from the ETS Research Data Repository (https://www.ets.org/research/contact/data_requests/).
